# Application of Machine Learning in the Reliability Evaluation of Pipelines for the External Anticorrosion Coating

**DOI:** 10.1155/2022/4759514

**Published:** 2022-03-24

**Authors:** Zhifeng Zhao, Mingyuan Chen, Heng Fan, Nailu Zhang

**Affiliations:** ^1^School of Electronic Engineering, Xi'an Shiyou University, Xi'an 710065, China; ^2^Dept of Mechanical, Industrial and Aerospace Engineering, Concordia University, Montreal H3G2W1, Canada

## Abstract

The purpose of this research is to enhance the analysis of the reliability status for external anticorrosive coatings. With the limitation and insufficiency of the static evaluation method, we study and construct an evaluation method of dynamic reliability for the anticorrosive layer, integrating the trend analysis of the Markov chain and the set pair theory. This method is implemented by the machine learning software of PyCharm community edition, based on *Python* language. The algorithm utilizes the connection degree in the set pair theory to determine the risk levels of the anticorrosive coating systems. According to the characteristics of the dynamic change of the anticorrosive layer with time, we built the mathematical evaluation model by combining it with the nonaftereffect property of the Markov chain. Therefore, we can make a dynamic and useful analysis for the reliability grade of the anticorrosive coating and assess the effectiveness grade of the changed reliability for the anticorrosive coating after some time. This method can effectively evaluate the reliability level of the anticorrosion coating through the example of big data of detection points. Under national standards, we provide the theoretical basis for pipeline maintenance within detection cycle requirements.

## 1. Introduction

The reliability evaluation of the pipeline external anticorrosive coating is a significant component of pipeline integrity management, which is related to the safety of pipeline energy transportation [1]. The current safety evaluation method mostly belongs to static evaluation. Because of the dynamic characteristics of the external anticorrosive coating, the static evaluation method cannot meet the dynamic requirements of the system in some aspects. Their numerical conclusions are often too simple to express danger. The poor expression of risk tendentiousness and dynamics is not conducive to providing decision-making services to managers [2, 3].

According to the research of reliability analysis methods at home and abroad, there are two main categories of external anticorrosion coating for pipelines: the evaluation method of direct safety and the evaluation method of mathematical theory analysis [4]. The evaluation methods of direct safety include the method of fault tree assessment, the safety checklist, the risk matrix diagram, the event tree analysis, the direct assessment method of external corrosion, and the manual of pipeline risk management. They belong to qualitative or semiquantitative methods. Generally, they can only give the risk levels or relative values, such as high, medium, or low. They have no unit and dimensional values or accident probability and have certain limitations. The analysis evaluation methods of applying mathematical theory include the analytic hierarchy process, the model method of BP neural network, the grey theory, and the fuzzy evaluation method. The model method of the BP neural network is to use the autonomous learning of neural network models and nonlinear approximations to obtain knowledge. It puts forward the ideas of serial-parallel processing and self-fault tolerance. However, the BP algorithm is only an improved method of nonglobal partial search. It requires that the solution is indeed the global extremum of a nonlinear function, which may cause the algorithm to fall into the local extremum and lead to training failure [5]. The analytic hierarchy process treats complex problems as systems. It decomposes the multiindex or constraint into various levels so as to evaluate and analyze the problems at various levels and find out the decision-making method of multiindex scheme optimization. However, if the amount of index data is too large, it is difficult to solve the eigenvalues and eigenvectors of the matrix, and the weight cannot be determined, resulting in the failure of the analysis. In the process of scheme formulation, the better one can only be selected from the known hierarchical schemes, but the shortcomings of the known schemes cannot be found, and the improvement methods cannot be put forward. So it cannot provide a new scheme for decision-making for evaluation. The fuzzy evaluation method of grey theory is to analyze data by using fuzzy mathematics and the grey system analysis method. However, the algorithms for parameter threshold and correlation degree in its membership function have obvious defects, which will have a great impact on the accuracy of the results [6, 7].

Aiming at the limitations and shortcomings of static evaluation, the dynamic evaluation of the deterioration trend of the anticorrosive coating based on the set pair (S.PA) and Markov chain theory is proposed [8, 9]. By using the connection degree of S.PA to determine the status of each deterioration grade in the anticorrosive coating, we established the reliability evaluation model of the degradation trend level of the anticorrosion layer [10, 11]. Combining the nonaftereffect and homogeneous of the Markov chain, the safety effectiveness of the anticorrosive coating varying at intervals of time is obtained according to the transfer matrix and modification [12, 13]. Through the above algorithm, we use the machine learning software of the PyCharm community edition based on the *Python* language to predict and intelligently analyze the detection data.

## 2. Methods

### 2.1. Set Pair Theory

Set pair theory uses connection degree to represent the connection component of two sets under the background of a certain problem. The connection degree is expressed by the normalized connection number. That is, *U*=*a*+*b*i+*cj*. “*a*” denotes the identity degree. “*b*” denotes the difference degree. That is, the difference is uncertain, and the characteristics are neither identical nor opposite in the two sets. “i” is a marker symbol or a difference coefficient. “*c*” is called the opposition degree. “*j*” is a marker symbol or a degree of opposition coefficient [14]. By using the connection formula of S.PA, we can use this method to describe the deterioration status of pipeline anticorrosive coating [15].

### 2.2. Markov Chain

It is a method proposed and named by the Russian mathematician Markov to analyze the stochastic process of things. It is different from other methods such as variance analysis and interval estimation. It has the characteristics of no aftereffect. Given the corrosion status of the pipeline anti-corrosive coating, the future corrosion status is only related to the corrosion status at the time of detection, which has the characteristics of the Markov chain. Therefore, the corrosion degradation process can be viewed as a Markov chain [16, 17].

For the pipeline coating, the transition probability between the degradation states of the coating is only related to the corrosion state. It has nothing to do with the time *n* of detection. Therefore, the corrosion state process can be regarded as a homogeneous Markov chain [18]. That is, in the case of any state *i*, *j* ∈ *S* = {1, 2,…, *n*} (state space), the transition probability *P*_*ij*_^(*n*)^ of the Markov chain is only related to state *i* and *j*. *P*_*ij*_^(1)^ is used to represent one-step transition probability, that is,

(1) $$\begin{array}{c} P_{ij}^{\left(1\right)}=\left(\begin{array}{cccc} P_{11} & P_{12} & \cdots & P_{1n}\\ P_{21} & P_{22} & \cdots & P_{2n}\\ \cdots & \cdots & \cdots & \cdots \\ P_{n1} & P_{n2} & \cdots & P_{nn} \end{array}\right), \end{array}$$

where *P*^(*n*)^=(*P*_*ij*_^(*n*)^) is n-step transition probability matrix, and *P*_*ij*_^(*n*)^ ≥ 0, ∑_*k*∈*s*_*P*_*ij*_^(*n*)^=1. The n-step transition probability *P*_*ij*_^(*n*)^ has the following properties [19]:

(2) $$\begin{array}{c} \begin{array}{c} P_{ij}^{\left(n\right)}=\underset{k\in S}{\sum }P_{ik}^{\left(l\right)}P_{kj}^{\left(n-l\right)},\\ \begin{array}{c} P_{ij}^{\left(n\right)}=\underset{k_{1}\in S}{\sum }\ldots \underset{k_{n-1}\in S}{\sum }P_{ik_{1}}P_{k_{1}k_{2}}\ldots P_{k_{n-1}j},\\ p^{\left(n\right)}=pp^{\left(n-1\right)},\\ p^{\left(n\right)}=p^{n}. \end{array} \end{array} \end{array}$$

At present, there are four methods for calculating the transition probability matrix, such as the solving method of the inverse matrix, the regression analysis method, the empirical judgment method, and the statistical analysis method.

#### 2.2.1. Statistical Analysis Method

The statistical analysis method calculates the percentage of one to another state in a certain period under the condition of data accumulation for many years. That is to say, the number *m*_*i*_ in the hypothesis “i” state is calculated. From “i” to “j” state, the transition number *m*_*j*_ is also calculated. The transition probability *P*_*ij*_^(1)^, *P*_*ij*_^(1)^=*m*_*j*_/*m*_*i*_ is used as the element of the matrix of transition probability, and then the matrix of transition probability is constructed in turn.

#### 2.2.2. Empirical Judgment Method

The empirical judgment method requires using the engineering experience to determine the transfer probability matrix. It is subjective and one-sided. The magnitude of each probability value is estimated by the subjective human. This method needs several years of verification and gradual revision before it tends to the actual state of the transfer law.

#### 2.2.3. Inverse Matrix Method

The inverse matrix method considers the inverse matrix to solve the transition probability matrix. It requires a few detection data. With the addition of new data, the transfer probability matrix can be amended to make the judged data results more reasonable and accurate. The method steps are as follows:

Assuming that the system has three states, the initial vector is *U*_1_={*a*_1_, *b*_1_, *c*_1_}, and the matrix of transition probability is

(3) $$\begin{array}{c} P=\left(\begin{array}{ccc} P_{11} & P_{12} & P_{13}\\ p_{21} & p_{22} & P_{23}\\ P_{31} & P_{32} & P_{33} \end{array}\right). \end{array}$$

Then the next year's value is *U*_2_={*a*_2_, *b*_2_, *c*_2_} and can be expressed as

(4) $$\begin{array}{c} U_{1}\times P=U_{2}. \end{array}$$

The equation is

(5) $$\begin{array}{c} a_{1}P_{11}+b_{1}P_{21}+c_{1}P_{31}=a_{2},\\ a_{1}P_{12}+b_{1}P_{22}+c_{1}P_{32}=b_{2},\\ a_{1}P_{13}+b_{1}P_{23}+c_{1}P_{33}=c_{2},\\ P_{11}+P_{12}+P_{13}=1,\\ P_{21}+P_{22}+P_{23}=1,\\ P_{31}+P_{32}+P_{33}=1. \end{array}$$

In the same way, the values of *U*_3_={*a*_3_, *b*_3_, *c*_3_} and *U*_4_={*a*_4_, *b*_4_, *c*_4_} in another two years can be listed into eight equations of the same form. The total set has twelve equations. The transition probability parameter solution has nine parameters. Therefore, four years of data can be used to solve the elements of the transition probability matrix in the system of equations, that is,

(6) $$\begin{array}{c} C\times P=D, \end{array}$$

(7) $$\begin{array}{c} C=\left(\begin{array}{ccc} a_{1} & b_{1} & c_{1}\\ a_{2} & b_{2} & c_{2}\\ a_{3} & b_{3} & c_{3} \end{array}\right), \end{array}$$

(8) $$\begin{array}{c} D=\left(\begin{array}{ccc} a_{2} & b_{2} & c_{2}\\ a_{3} & b_{3} & c_{3}\\ a_{4} & b_{4} & c_{4} \end{array}\right). \end{array}$$

The transition probability matrix is

(9) $$\begin{array}{c} P=C^{-1}\times D. \end{array}$$

#### 2.2.4. Regression Analysis

Regression analysis is based on the assumption that the transfer probability distribution obeys a certain distribution law. For example, it follows a normal distribution and then obtains the probability distribution of the estimated value. The first step requires establishing the regression equation between the variables (regression accuracy W). The next step is to determine *M*_*i*_ (the median value of state i) into the regression equation and inversely calculated *t*_*i*_ (number of years). Then *t*_*i*+1_ is substituted into the regression equation, calculated the expected value for the next year and assumed that the transition probability also obeys the normal distribution. According to the expected value, we determine the standard deviation (accuracy of the regression equation) and the discrete state distribution. So, we determine the probability distribution of each state, and construct the transition probability matrix P. From the above calculation steps, we observed that the results are to be verified by assuming that the distribution law of transition probability obeys normal distribution. Then the regression model is checked, and the errors are calculated and predicted.

Establishment steps of a dynamic assessment model for the deterioration tendency grade of external anticorrosion coating.

According to the evaluation standard of external anticorrosion coating, we select reasonable performance parameters that can reflect the damage and deterioration level of the anticorrosion coating and define the deterioration level of the external anticorrosion coating. We establish the evaluation model of connection degree by using the set pair theory.

The initial state distribution of the anticorrosion coating system is established by using the detection data of the external anticorrosion coating in different years, and the solution method of the appropriate transfer probability matrix is selected to calculate the transfer probability matrix.

Combined with the properties of the Markov chain, we establish the dynamic evaluation model of the Markov chain of the set pair theory by using the transition probability matrix and initial state distribution. Therefore, we evaluate the distribution of deterioration states of the external anticorrosion coating [20].

Application of dynamic assessment model for the deterioration tendency grade of external anticorrosive coating.

The actual detection methods and data of gas and oil pipelines in the project site allow us to select the breakage points density classification of the anticorrosive coating as the evaluation index. At present, the methods and technologies to judge the damage point of anticorrosion coating are as follows: numerical direct reading method, radiation distance method, statistical graphics method, voltage-controlled vibration frequency method, cursor observation method, and magnetic field drop method [21, 22]. With the actual requirements of maintenance measures, the deterioration level of external anticorrosive coating is divided into three levels: the first level is classified as the excellent and good status of the damage point density; the second level is a normal status of the damage point density; and the third level is classified as a bad status of the damage point density [23, 24].  Table 1 shows the classification standard for the degradation of the external anticorrosive coating. “Bp” is the density value of the breakage point of the anticorrosive coating. The minimum distance between two adjacent damaged points is not more than twice the central burial depth of the pipeline, which can be marked as one.

According to the connection degree of the S.PA, a definition is introduced: *U*_*m*_=*a*+*bi*+*cj*, m = (1, 2,…, n) (natural number). “a” represents the first-class grade of excellent and good anticorrosive coating in the whole pipeline. “b” indicates the second-class grade of normal anticorrosive coating in the whole pipeline. “c” shows the third-class grade of bad anticorrosive coating in the whole pipeline. “i” represents the marker of the normal grade of pipeline. “j” indicates the marker of the bad grade and opposition to the excellent and good grade. As an example of the Bp data of breakage points of asphalt anticorrosive coating for the 6909-meter pipeline, we calculate that the pipeline length with excellent and good anticorrosive coating is 4421m, so *a* = 4421/6909 = 0.6399. Similarly, the pipeline length in the second grade is 2211m, so *b* = 2211/6909 = 0.3200. The pipeline length in the third-grade is 277m, so *c* = 277/6909 = 0.0401.  Table 2 describes the classification status summary of inspection data collated in different periods for the anticorrosive coating (detection cycle every three years). Therefore, the initial state distribution model for the anticorrosive coating pipeline is *U*_1_=0.6399+0.3200*i*+0.0401*j*. In other years, the state distribution models of the anticorrosive coating pipeline are as follows:

(10) $$\begin{array}{c} U_{2}=0.4198+0.4101i+0.1701j,\\ U_{3}=0.3198+0.4100i+0.2702j,\\ U_{4}=0.2698+0.4000i+0.3302j,\\ U_{5}=0.2451+0.3901i+0.3648j. \end{array}$$

We observed the state model that the deterioration of coating becomes more and more obvious. That is, the changing trend of the good grade is from 0.6399 to 0.2451. With the increase of service years, the good grade becomes smaller. The changing trend of the normal grade is from 0.3200 to 0.3901. By comparing the indexes nearly four times, we found that the changing trend of normal grade is about 0.40, which conforms to the basic change law of the pipeline life cycle. The changing trend of the bad grade is from 0.0401 to 0.3648, and the 4th deterioration state of coating has begun to be greater than that of the good grade (0.3302 > 0.2698). The trend of aging is obvious.

The same conclusion can be drawn from the set-pair tendency. When the set-pair tendency is used for the reliability system evaluation, the set-pair tendency represents the reliability tendency. Its reliability analysis is based on the hierarchical relationships in the connection number. Taking the ternary connection number as an example, it indicates the degree to which the relationship between (*a*, *b*) and (*c*) affects the deterioration development. That is, ∂*c*=*c*/(*a*+*b*), which indicates the dynamic development of the deterioration direction. In the example, the deterioration level of external anticorrosion coating for the pipeline has developed from 0.0401/(0.6399 + 0.3200) = 0.0418 to 0.3648/(0.2451 + 0.3901) = 0.5743, and the degree of deterioration is obviously large.

According to the analysis of the data characteristics of Table 2, we selected the inverse matrix method to solve the transition probability matrix. Therefore, the machine learning software of PyCharm community edition is used for data analysis. We construct the matrix *C*_1_ and *D*_1_ by the four distribution vectors corresponding to the detection data of the previous four times. That is,

(11) $$\begin{array}{c} C_{1}=\left[\begin{array}{c} \begin{array}{cc} \begin{array}{cc} 0.6399 & 0.3200 \end{array} & 0.0401 \end{array}\\ \begin{array}{cc} 0.4198 & \begin{array}{cc} 0.4101 & 0.1701 \end{array} \end{array}\\ \begin{array}{cc} 0.3198 & \begin{array}{cc} 0.4100 & 0.2702 \end{array} \end{array} \end{array}\right],\\ D_{1}=\left[\begin{array}{c} \begin{array}{cc} 0.4198 & \begin{array}{cc} 0.4101 & 0.1701 \end{array} \end{array}\\ \begin{array}{cc} 0.3198 & \begin{array}{cc} 0.4100 & 0.2702 \end{array} \end{array}\\ \begin{array}{cc} 0.2698 & \begin{array}{cc} 0.4000 & \;0.3302 \end{array} \end{array} \end{array}\right]. \end{array}$$

According to equation (2)–(9), we calculate the transfer probability matrix *P*_1_ through python programming based on the previous four detection data,

(12) $$\begin{array}{c} P_{1}=\left[\begin{array}{c} \begin{array}{cc} \begin{array}{cc} 0.5642 & 0.3684 \end{array} & 0.0674 \end{array}\\ \begin{array}{cc} 0.1756 & \begin{array}{cc} 0.5112 & 0.3132 \end{array} \end{array}\\ \begin{array}{cc} 0.0643 & \begin{array}{cc} 0.2686 & 0.6671 \end{array} \end{array} \end{array}\right], \end{array}$$

with the properties of the Markov chain, we evaluate the deterioration value of anticorrosive coating in June 2021,

(13) $$\begin{array}{c} U_{5}^{\prime }=\left[\begin{array}{ccc} 0.2437 & 0.3926 & 0.3637 \end{array}\right]. \end{array}$$

Compared with the fifth detection value in August 2021, there exists a certain deviation. Therefore, we modify the transition probability matrix.

Firstly, we establish four distribution vectors corresponding to the last four detection data. We construct the matrices *C*_2_ and *D*_2_, which is

(14) $$\begin{array}{c} C_{2}=\left[\begin{array}{c} \begin{array}{cc} 0.4198 & \begin{array}{cc} 0.4101 & 0.1701 \end{array} \end{array}\\ \begin{array}{cc} 0.3198 & \begin{array}{cc} 0.4100 & 0.2702 \end{array} \end{array}\\ \begin{array}{cc} 0.2698 & \begin{array}{cc} 0.4000 & 0.33 \end{array} \end{array}02 \end{array}\right],\\ D_{2}=\left[\begin{array}{c} \begin{array}{cc} 0.3198 & \begin{array}{cc} 0.4100 & 0.2702 \end{array} \end{array}\\ \begin{array}{cc} 0.2698 & \begin{array}{cc} 0.4000 & 0.3302 \end{array} \end{array}\\ \begin{array}{cc} 0.2451 & \begin{array}{cc} 0.3901 & 0.3648 \end{array} \end{array} \end{array}\right]. \end{array}$$

Secondly, according to equation (2)–(9), we calculate the transfer probability matrix *P*_2_ through python programming based on the last four detection data,

(15) $$\begin{array}{c} P_{2}=\left[\begin{array}{c} \begin{array}{cc} \begin{array}{cc} 0.6223 & 0.2658 \end{array} & 0.1119 \end{array}\\ \begin{array}{ccc} 0.0921 & 0.6587 & 0.2492 \end{array}\\ \begin{array}{ccc} 0.1223 & 0.1663 & 0.7114 \end{array} \end{array}\right]. \end{array}$$

Finally, *P*_1_ is used to take the mean value of *P*_2_ to modify the transition probability matrix, and *P*_*mo*  *d*_ is calculated by the python program,

(16) $$\begin{array}{c} P_{mo\;d}=\left[\begin{array}{c} \begin{array}{cc} \begin{array}{cc} 0.5933 & 0.3171 \end{array} & 0.0896 \end{array}\\ \begin{array}{ccc} 0.1339 & 0.5849 & 0.2812 \end{array}\\ \begin{array}{ccc} 0.0933 & 0.2175 & 0.6892 \end{array} \end{array}\right]. \end{array}$$

According to the modification of the transfer probability matrix, the deterioration value of the anticorrosive coating by the *Python* program algorithm in August 2024 is predicted as follows:

(17) $$\begin{array}{c} U_{6}=0.2317+0.3852i+0.3831j. \end{array}$$

Similarly, the deterioration value of the anticorrosive coating in August 2027 is predicted as follows:

(18) $$\begin{array}{c} U_{7}=0.2248+0.3821i+0.3931j. \end{array}$$

**Figure 1:**
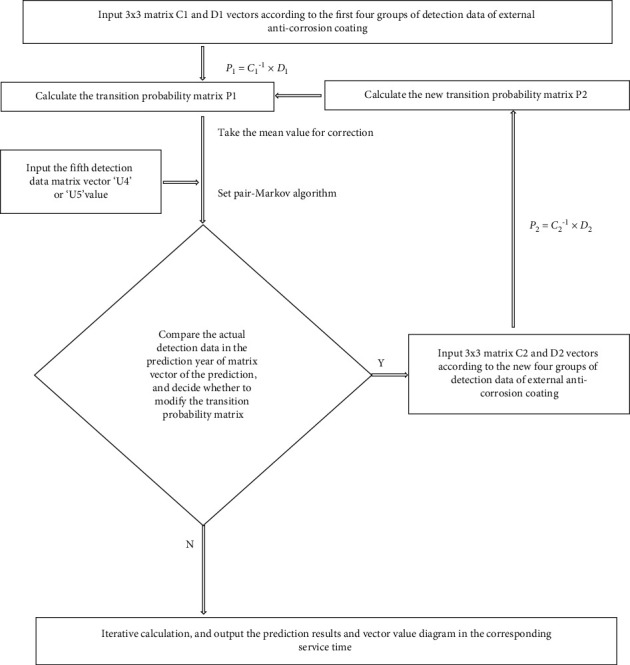
The python flow chart of the set pair-Markov algorithm.

**Figure 2:**
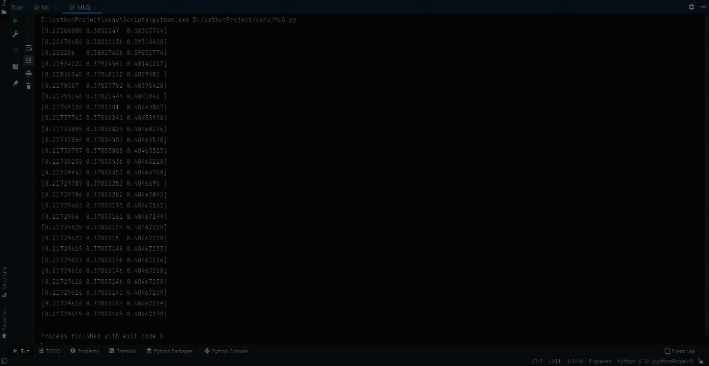
The *Python* calculation value of the set pair-Markov algorithm for the external anticorrosion coating of the pipeline.

**Figure 3:**
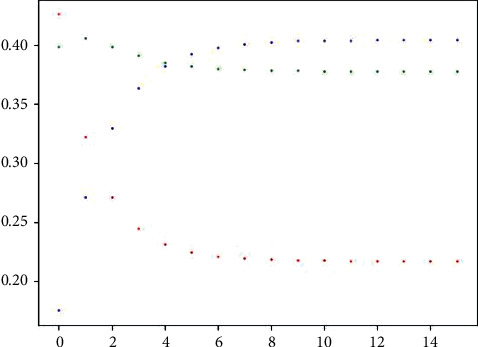
The iterative value for predicting the deterioration of the external anticorrosion coating of pipeline against the set pair-Markov matrix vector.

The above flow chart of the *Python* program is shown in the *Python* flow chart of the set pair-Markov algorithm in Figure 1.  Figure 2 is the python calculation value of the set pair-Markov algorithm for the pipeline's external anticorrosion coating. The data vector iterates through the set pair-Markov algorithm until the product of the vector remains unchanged, and outputs the result.  Figure 3 is the iterative value diagram for predicting the deterioration of the external anticorrosion coating of the pipeline against the set pair-Markov matrix vector. The red dot represents the iterative value distribution of pipeline tendency in excellent and good condition. The green dot represents the iterative value distribution of pipeline tendency in the general state. The blue dot represents the iterative value distribution of pipeline tendency in a poor state.

## 3. Results

In August 2024, the first class of the anticorrosion layers will reach 23.17%, the second class will reach 38.52%, and the third class will reach 38.31%. In August 2027, the deterioration resulting in anticorrosion coating will have 22.48% in the first class, the second class will have 38.21%, and the third class will have 39.31%. From 2024 to 2027, the deterioration tendency further increases, and the poor state of the anticorrosion coating is nearly twice that of the good state. The degree of deterioration is larger. Through program calculation, it is found that the transition probability matrix P will basically not change and the limit matrix B will appear when *m* ≥ 11. That is, B $=\left[\begin{array}{c} \begin{array}{cc} \begin{array}{cc} 0.2173 & 0.3780 \end{array} & 0.4047 \end{array}\\ \begin{array}{cc} \begin{array}{cc} 0.2173 & 0.3780 \end{array} & 0.4047 \end{array}\\ \begin{array}{cc} \begin{array}{cc} 0.2173 & 0.3780 \end{array} & 0.4047 \end{array} \end{array}\right]$. For steady-state, it is worth noting that the product of the transition matrix and steady-state must be steady-state again. When the limit matrix B is obtained, the steady-state distribution probability of the quality status of the anticorrosion coating for the whole pipeline can be obtained. The steady-state distribution probability *π* = [0.2173, 0.3780, 0.4047]. It shows that the steady-state distribution probability is independent of the initial vector U4 and only related to the limit matrix B. It can be seen that the quality distribution will tend to be stable when the inspection times of the anticorrosion coating for the pipeline are enough. The proportion of pipeline sections with a quality grade of excellent and good is 21.73%. The proportion of pipeline sections with a general quality grade is 37.80%. The proportion of pipeline sections with a poor-quality grade is 40.47%. The excellent and good condition is maintained at one fifth of the pipeline. The other two conditions account for two fifths each. The deterioration tendency is obvious. We need to take maintenance measures to prevent the deterioration of the anticorrosion coating.

## 4. Discussion

With the development of the corresponding detection technology for the pipeline, the accuracy and statistical management of coating detection data will be more meticulous in the improvement and standardization of the relevant standards. Meanwhile, the methods of modifying the transfer matrix are also increasing, such as the arithmetic average method, weighted arithmetic average method, geometric average method, weighted geometric average method, square average method, and the harmonic average method. The transfer probability matrix P can be updated and revised in time, making the evaluation results more in line with the actual situation on the spot. So, we will grasp more accurately the deterioration trend of the anticorrosion layer. It has great significance for prolonging the service life of pipelines.

## 5. Conclusions

Through the case analysis, we evaluate the deterioration status of the anticorrosive coating in 2024 and 2027 and judge the safety grade of the anticorrosive coating comprehensively. The evaluation results can reflect the reliability status of the anticorrosive coating of pipelines in a timely, objective, and comprehensive manner. It makes up for the limitations and shortcomings of static evaluation. It also provides a decisive theoretical basis for pipeline maintenance.

If the corresponding maintenance measures are taken for the normal and bad anticorrosion coatings, the reliability and quality level of the anticorrosion coating will be improved. But the deterioration of the anticorrosion coating will continue to deteriorate without any maintenance measures. This is a dynamic characteristic of the damage distribution of the external anticorrosion coating. Combining with the machine learning software of PyCharm community edition based on the *Python* language, the intelligent detection data analysis is carried out by taking advantage of its simplicity, wide application fields, high development efficiency, and comprehensive class library. Through the set pair theory with the Markov chain, we can effectively analyze the change, and evaluate the distribution of deterioration trend of pipeline coating. According to the actual situation of pipelines, each pipeline company can guide and formulate the corresponding evaluation and detection times for the three to eight-year cycles in the national standards and integrate them. The optimization of pipeline anticorrosive coating is guaranteed under reasonable safety and economic conditions. It also provides a basis for the pipeline maintenance of formulating scientific and efficient schemes.

## Figures and Tables

**Table 1:** Classification standard for the degradation of the external anticorrosive coating.

Type of external anticorrosive coatings	Degradation status classification			
Asphalt anticorrosive coating, anticorrosive insulation layer of rigid polyurethane foam	Level I		Level II	Level III
	1 (excellent)	2 (good)	3 (normal)	4 (Bad)
	Bp ≤ 0.2	0.2 < Bp < 1	1≤Bp ≤ 2	Bp > 2

**Table 2:** Summary table of inspection data for the coating classification status.

Number of detections	Level		
	Level I (excellent and good)	Level II (normal)	Level III (bad)
1 (2009.06)	0.6399	0.3200	0.0401
2 (2012.07)	0.4198	0.4101	0.1701
3 (2015.07)	0.3198	0.4100	0.2702
4 (2018.06)	0.2698	0.4000	0.3302
5 (2021.08)	0.2451	0.3901	0.3648

## Data Availability

The raw data is obtained through the pipeline breakage points collected every three years in the third-party detection department of PetroChina Changqing Oilfield Company. The raw data required for these findings cannot be shared at this time as the data also forms part of an ongoing study.
